# A programmable benchtop photocrosslinking chamber for controlled bioconjugation

**DOI:** 10.1016/j.ohx.2026.e00804

**Published:** 2026-06-15

**Authors:** Samuel Olson, Gulsu Sener, Alan Weisgerber, Kai Tao

**Affiliations:** Cancer Early Detection Advanced Research Center (CEDAR), Knight Cancer Institute, Oregon Health & Science University, Portland, OR, USA

**Keywords:** Bioconjugation, Photochemistry, Ultraviolet, Photocrosslinking, Photoreactor

## Abstract

Ultraviolet (UV) photochemical reactions have become ubiquitous in the biomedical sciences, where they are frequently used to conjugate biomolecules in a process known as photo-crosslinking. Such processes play an important role in the fields of drug discovery, biomarker validation, and bioprinting. Typically, these photochemical reactions are conducted using commercially available UV irradiation sources which are bulky and expensive. Handheld units are a popular substitute but tend to cause excessive heating of the sample. Additionally, currently available UV sources are not designed to be user-friendly for performing bioconjugation in Eppendorf tubes, making them less practical for efficient and precise biomedical experiments. Here we present an open-source, low-cost (approximately $250), and customizable UV irradiation system that allows for simultaneous processing of up to six 1.5 mL Eppendorf tubes. Our system uses uniformly arranged 365 nm UV LEDs that irradiate the bottom of the tube with a polished aluminum sleeve for even distribution of light throughout the tube volume, resulting in higher efficiency and a 50% reduction in sample heating compared to commercial mercury lamp blanket exposure systems. The modular design allows LEDs of other wavelengths to be easily installed for broader photoreaction applications. Using this user-friendly system, we demonstrate the efficient conjugation of photocrosslinkable nanobodies to antibodies in 5 min without causing photobleaching of the fluorophores, thus enabling immunofluorescent imaging for biomedical applications.

## Graphical abstract

**Figure:**
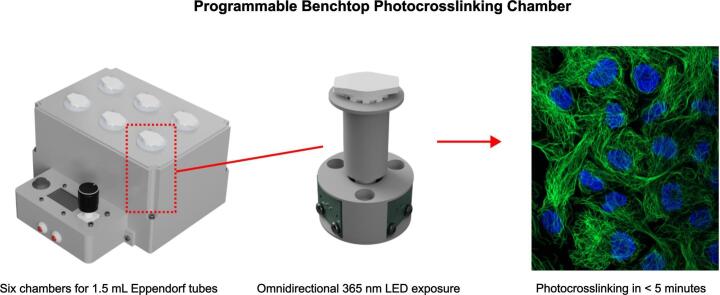

Specifications table

**Table:** 

Hardware name	A portable, efficient photochemical reaction system
Subject area	Biological sciences
Hardware type	Biological sample handling and preparation
Closest commercial analog	Sigma Aldrich SynLED Parallel Photoreactor; AlphaThera LED PX2 Hoefer UVC500; Crystal Technology BG-40A; Agiltron Inc. UVCG-203111111; Rayonet RPR-100
Open source license	This work is licensed under a Creative Commons Attribution 4.0 International License.
Cost of hardware	$250
Source file repository	https://doi.org/10.17632/v5yj8g5xts.1

## 1 Hardware in context

Photoreactive crosslinking is a process that involves the use of ultraviolet (UV) light to initiate chemical reactions forming covalent bonds between two molecules. These reactions have a wide range of applications in the biomedical sciences where they are frequently used for conjugation of biomolecules including proteins [1], [2], [3], [4], peptides [5], [6], and DNA/RNA [7], [8], [9], [10], [11], [12], [13]. These photoactivated bioconjugation strategies play a critical role in elucidating structure–function relationships, mapping protein–protein and protein-RNA interactions, and have applications in the field of drug delivery [8], [9]. Typically, the sample to be crosslinked is placed in a UV-transparent tube or plate and irradiated for a set duration at a set power output to yield a specific energy input into the material that allows the photochemical reaction to take place. Reactants, especially proteins and RNA, are highly sensitive to temperature and photodamage, and must retain functional integrity post-exposure. Therefore, the energy input must be low enough, or there must be a cooling methodology employed, to maintain a biologically compatible temperature range. In practice, most photocrosslinking experiments in biomedical laboratories are performed in standard Eppendorf microcentrifuge tubes, however, currently available UV systems are not optimized for efficient, uniform, and reproducible bioconjugation in such vessels. Moreover, emerging applications in areas such as 3D bioprinting and biomaterials engineering further highlight the need for controlled and reproducible photochemical processes [14], [15], [16].

Many commercially available UV photo-crosslinking systems use mercury lamps as a source due to their high power output. However, this power tends to be used inefficiently in a “blanket expose” method where samples are placed in the chamber (in the case of systems specifically designed for UV photo-crosslinking as well as general systems such as nail lamps), or directly on the source (in the case of handheld units) before being exposed to high-power UV. Due to the high power output of these systems, passive cooling in the form of an ice bath or active cooling in the form of a Peltier cooler are typically employed to reduce the temperature of the sample during irradiation. Chen et al. investigated epitope-directed antibody selection using site-specific photocrosslinking. In their study, samples needed to be plated on ice during the irradiation with a 6 W 365 nm UV light until complete reaction was achieved [17].

More recently, UV light emitting diodes (LEDs) have emerged as an alternative to traditional mercury lamps. They boast higher efficiency, consistent light intensity, and lower sample heating [18], [19]. The power output of UV LEDs tends to be lower than mercury lamps, but they are more efficient due to their focused viewing angle compared to the isotropic nature of mercury lamp emission. They are also more environmentally sustainable, as mercury lamps pose potent public and environmental health risks [20], [21]. Despite these advantages, commercially available UV LED-based photoreactors remain costly and are often not tailored for routine benchtop biomedical workflows. These devices also tend to use higher-power UV LEDs, resulting in increased process temperatures. In particular, there remains a need for a practical, low-cost system that enables rapid, reproducible, and thermally controlled photocrosslinking in standard biomedical lab settings. This need is especially urgent in antibody labeling using photocrosslinkable moieties, where maintaining protein integrity and minimizing photodamage on the probes or drugs are critical [22], [23]. Hui et al. investigated light-activated, site-specific conjugation of native IgGs using a 365 nm UVP CL-1000 UV crosslinker, a commercial instrument costing several thousand dollars. To mitigate heat-induced damage to antibody samples, the photocrosslinking reactions were conducted in an ice bath [24].

Here we introduce a low-cost, low-power UV photoreactor for use with standard Eppendorf microcentrifuge tubes, enabled by precise positioning of LEDs close to the tube body. This system is optimized for uniform and controlled photocrosslinking, enabling reproducible bioconjugation under biologically compatible conditions. We further demonstrate its utility in antibody bioengineering through the efficient photocrosslinking of nanobody-based systems, highlighting its potential for broad applications in biomedical research.

**Fig. 1:**
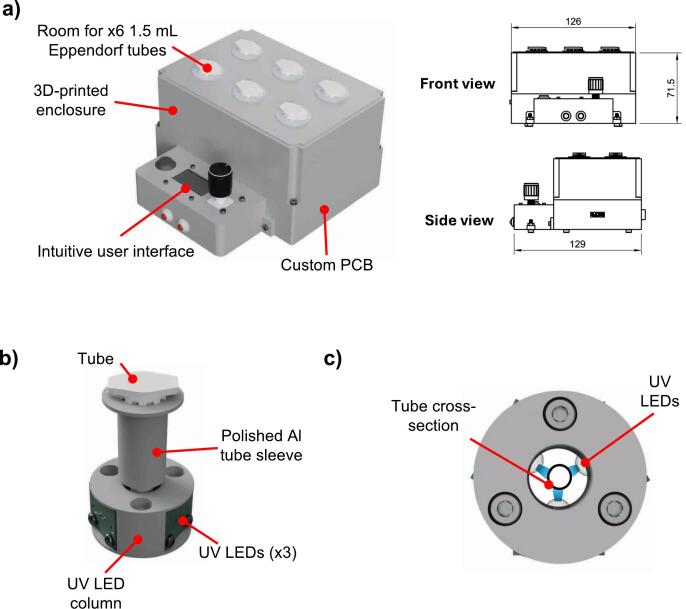
Overview of the UV LED system. a) Render and drawing of the assembled system (dimensions in mm), b) render of UV LED column, showing placement of 1.5 mL Eppendorf tube and polished tube sleeve for maximizing UV reflectance, c) top-down view of UV LED column demonstrating LED placement.

An overview of the system is shown in Fig. 1a. The enclosure is made entirely out of 3D-printed components and allows for parallel photo-crosslinking experiments in up to six 1.5 mL Eppendorf tubes simultaneously. Fig. 1b shows one of six individual UV LED column units, wherein the bottom of the tube is exposed to 365 nm UV light from three UV LEDs. The polished aluminum tube sleeve allows reflectance of off-axis UV light to be redirected into the tube. The top-down view in Fig. 1c shows the circular arrangement of the UV LEDs placed at 120° intervals surrounding the cross-section of the tube, allowing for uniform exposure of the sample. The operation of the LEDs is controlled by a custom PCB based around an Arduino Nano microcontroller, complete with a user interface for selecting exposure time and the quantity of tubes to process. The system is a fraction of the cost of similar commercial systems, and it can be constructed with just a 3D printer, a laser cutter, and a soldering iron (Table 1).

**Table 1:** Key components of the system (see bill of materials for complete list and source URLs).

**Supplier**	**Part Number**	**Description**	**Number**	**Cost per unit (USD)**	**Total cost (USD)**
Arduino	A000005	Arduino Nano	1	21.17	21.17
Digi-Key	1125–1509-ND	365 nm low-power LEDs	18	4.89	88.02
Digi-Key	CL2N3-G-ND	20 mA LED driver	6	0.41	2.46
Amazon	B07F26CT6B	Rotary encoder	1	2.00	2.00
Amazon	B09C5K91H7	OLED display	1	3.00	3.00
Digi-Key	563–1108-ND	17 mm fan	1	11.60	11.60

## 2 Hardware description

The hardware presented in this work is a compact, low-cost, easy to use system for performing photochemical reactions. The use of UV LEDs instead of mercury lamps leads to increased efficiency, more consistent power delivery, and lower sample heating over most commercially available units. The system is designed to accommodate 1.5 mL Eppendorf tubes, which slide into a custom polished aluminum sleeve and are held in a position for optimal exposure to the UV LED array. Although the version presented here employs 365 nm LEDs, it is straightforward to swap in LEDs of other wavelengths due to the modular design of the system.

A few highlights of the system are listed below:

• Total power density of 450 W/m^2^ per column
• 50% reduction in sample heating over mercury lamp-based systems
• Low cost, easy to build, and easy to use, facilitating implementation in a wide breadth of labs
• Easily customizable with LEDs of various wavelengths
• Ability to process up to 6 samples simultaneously

### 2.1 System overview

**Fig. 2:**
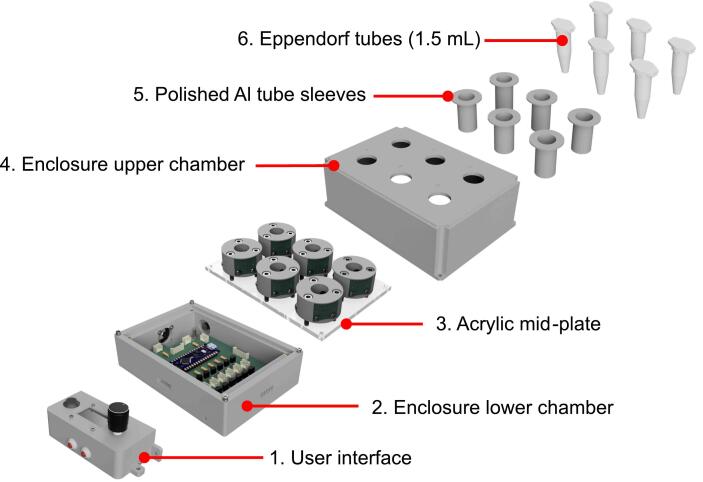
Exploded view of the system showing the main components.

The system consists of six main components, as shown in the exploded diagram of Fig. 2. The user interface (1) consists of a 3D-printed enclosure that houses the power switch, an OLED screen for displaying the parameter selection menu, a rotary encoder knob for selecting parameters, a green power indicator LED which illuminates when the power to the system is activated, and a red “active UV” indicator LED that illuminates when the tubes are being processed (i.e., when UV light is being emitted). This enclosure is then attached to the lower chamber of the main enclosure (2), which is also 3D-printed. The main enclosure houses the custom PCB as well as a small 17 mm fan and power jack. Attached to the lower chamber is the laser-cut acrylic mid-plate (3), which holds the six UV LED columns. To seal the enclosure, the 3D-printed upper chamber (4) is attached to the lower chamber. The upper chamber has six openings for holding the polished aluminum tube sleeves (5), which in turn hold 1.5 mL Eppendorf tubes (6). The tubes are held in a position such that the lowest portion of the tube coincides with the center point of the exposure angle of the UV LEDs.

### 2.2 Electrical components

**Fig. 3:**
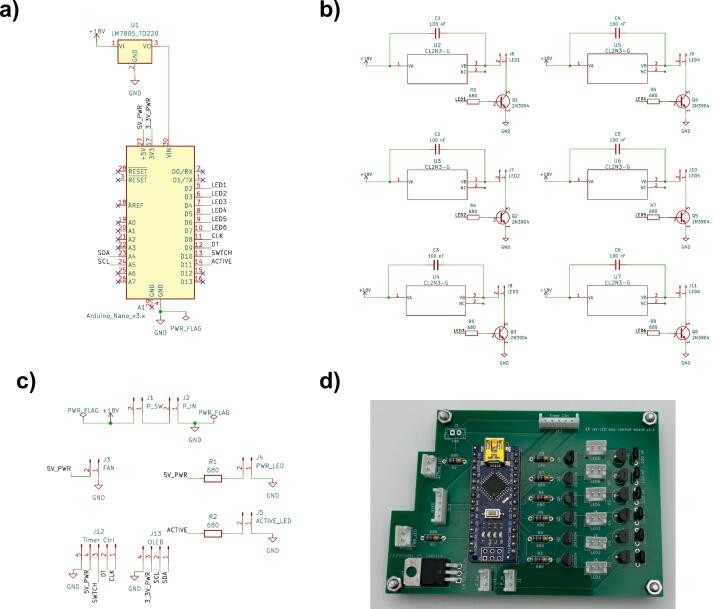
a) Schematic of microcontroller circuit, b) schematic of LED power circuits, c) schematic of user interface circuits, d) assembled printed circuit board.

The user interface and the output of the UV LEDs is controlled via an Arduino Nano microcontroller (Fig. 3a). Each UV LED column is switched on or off using a 2N3904 transistor switching circuit connected to digital I/O pins on the Arduino (Fig. 3b). The current through the LEDs is regulated to 20 mA by a constant current driver (Microchip Technology CL2N3-G) with a 100 nF smoothing capacitor. The constant current protects the LEDs, while also providing consistent UV LED power to the sample. This current is supplied to each UV LED column from the circuit board via a detachable JST connector for ease of disassembly. Power input, indicator LEDs, and the user interface are also connected via JST connectors (Fig. 3c). A custom PCB was designed and manufactured for ease of assembly (Fig. 3d), however the components can also easily be soldered onto perfboard to further reduce costs.

**Fig. 4:**
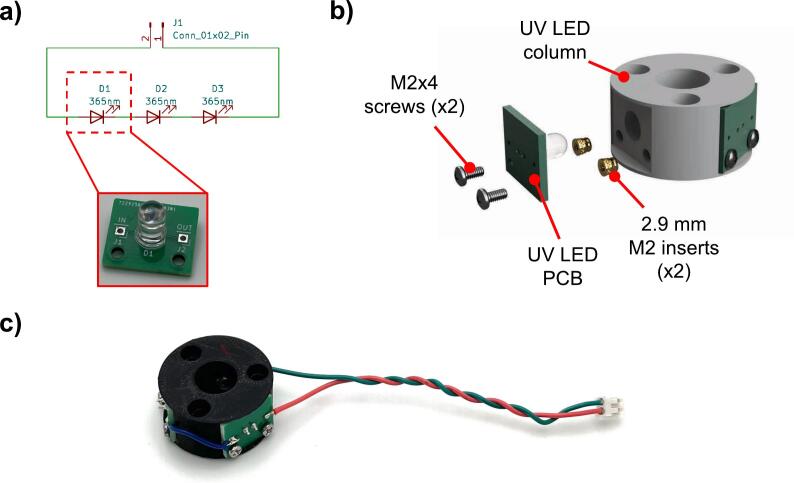
a) Schematic of UV LED sub-circuit with inset showing assembled individual PCB, b) attaching the UV LED PCBs to the UV LED column, c) assembled and wired UV LED column subassembly with JST connector.

Each UV LED column consists of three 365 nm wavelength LEDs with a nominal power output of 5.5 mW each. The LEDs are wired in series and connected to a JST connector (Fig. 4a). Each LED is soldered onto a small PCB for consistent placement on the column (inset, Fig. 4a). Since the LEDs are current controlled from the main PCB, the 365 nm LEDs on the sub-PCB can be swapped for low-power LEDs of other wavelengths (e.g., 395 nm, 405 nm, etc.) based on user needs without needing to consider forward voltage. The UV LED PCBs are attached to the 3D-printed UV LED column via M2x4mm screws that thread into heat-set M2 inserts (Fig. 4b). The PCBs are wired in series via 25 mm jumper wires, and the circuit is connected to the PCB via a JST connector wired to the positive side of D1 (18 V) and the negative side of D3 (GND), as in Fig. 4a. An assembled column is shown in Fig. 4c. The wired connectors are passed through holes in the acrylic mid-plate and plugged into receptacles on the main PCB.

### 2.3 Firmware

The system’s firmware consists of a single, simple file written in Arduino C++. Variables are provided for adjusting the total number of LEDs in the system (variable “TOT_LEDS”, default 6), the maximum exposure time allowed in seconds (variable “MAX_TIME”, default 600), and the time increment resolution in seconds (variable “TIME_DELTA”, default 15).

### 2.4 System applications

The following points highlight the importance of this hardware for enabling and improving laboratory tasks:

• **Focused UV LED irradiation** provides the ability to perform low temperature photocrosslinking of sensitive biological material
• **Six 1.5 mL Eppendorf tube slots** allow for parallel processing of multiple samples in standard laboratory vessels
• **Ease of changing exposure wavelength** enables a wider breadth of photocrosslinking experiments to be performed using this system
• **Polished aluminum tube sleeves** allow for uniform exposure across the surface area of tube

## 3 Design files summary

**Table:** 

**Design file name**	**File type**	**Open source license**	**Detail**	**Location**
controls_housing.step	STEP	CC BY 4.0		Mendeley Data: https://doi.org/10.17632/v5yj8g5xts.1
enclosure_lower_chamber.step	STEP	CC BY 4.0		Mendeley Data: https://doi.org/10.17632/v5yj8g5xts.1
enclosure_upper_chamber.step	STEP	CC BY 4.0		Mendeley Data: https://doi.org/10.17632/v5yj8g5xts.1
led_column.step	STEP	CC BY 4.0		Mendeley Data: https://doi.org/10.17632/v5yj8g5xts.1
midplate.dxf	DXF	CC BY 4.0		Mendeley Data: https://doi.org/10.17632/v5yj8g5xts.1
main_board_pcb.zip	Zipped Gerber	CC BY 4.0		Mendeley Data: https://doi.org/10.17632/v5yj8g5xts.1
uv_led_sub_board_pcb.zip	Zipped Gerber	CC BY 4.0		Mendeley Data: https://doi.org/10.17632/v5yj8g5xts.1
main_board_schem.pdf	PDF	CC BY 4.0	−	Mendeley Data: https://doi.org/10.17632/v5yj8g5xts.1
uv_led_sub_board_schem.pdf	PDF	CC BY 4.0	−	Mendeley Data: https://doi.org/10.17632/v5yj8g5xts.1
uv_photoreactor_firmware.ino	Arduino	CC BY 4.0	−	Mendeley Data: https://doi.org/10.17632/v5yj8g5xts.1

• *controls_housing.step*: 3D-printed enclosure that houses user interface controls including the power switch, power LED, active LED, OLED screen, and the rotary encoder.
• *enclosure_lower_chamber.step*: 3D-printed enclosure for the lower chamber of the system. This enclosure houses the main PCB, power input jack, cooling fan, and the mid-plate.
• *enclosure_upper_chamber.step*: 3D-printed enclosure for the upper chamber of the system. This enclosure attaches to the lower enclosure and supports the polished aluminum sleeves that hold the sample tubes.
• *led_column.step*: 3D-printed fixture on which three UV LED sub board PCBs are mounted. This column is then in turn mounted to the mid-plate.
• *midplate.dxf*: acrylic mid-plate of the system on which the LED columns are placed. This piece is laser cut from 1/8″ thick acrylic stock.
• *main_board_pcb.zip*: Gerber files for the main PCB which contains the Arduino microcontroller, transistor switches, user interface input/outputs, and LED current control.
• *uv_led_sub_board_pcb.zip*: Gerber files for the LED sub-board which contains a single LED and is mounted to the LED column
• *main_board_schem.pdf*: Schematic showing electrical connections on the main board
• *uv_led_sub_board_schem.pdf*: Schematic showing electrical connections on the LED sub-board
• *uv_photoreactor_firmware.ino*: Arduino firmware for the system

## 4 Bill of materials summary

The bill of materials is available in the following Mendeley data repository: https://doi.org/10.17632/v5yj8g5xts.1.

## 5 Build instructions

Prior to beginning the build, purchase all necessary items based on the bill of materials located in the corresponding Mendeley data repository.

### 5.1 3D-printing, laser cutting and PCB acquisition

1. 3D-print components with 20% infill PLA (Polymaker #PA07001) using a 0.2 mm nozzle at 215 °C and a heated bed temperature of 60 °C
  a. *controls_housing.step*
  b. *enclosure_lower_chamber.step*
  c. *enclosure_upper_chamber.step*
  d. *led_column.step*
2. Laser cut *midplate.dxf* from 1/8″ acrylic stock (McMaster-Carr #8560 K239) using a laser power of 45 W and a cutting speed of 15 mm/sec
3. Obtain PCBs from your choice of PCB manufacturer or cut perfboards to size if not using PCB
  a. If using PCB, order:
    i. x1 main PCB according to Gerber files in main_board_pcb.zip
    ii. x18 UV LED sub-board PCBs according to Gerber files in uv_led_sub_board_pcb.zip
  b. If using perfboard, cut:
    i. x1 100 mm by 70 mm section for the main board
    ii. x18 16 mm by 14 mm sections for the LED sub-boards
    iii. Drill mounting holes according to the locations in the PCB layout

### 5.2 Main circuit board

1. Solder x1 Arduino Nano (Arduino #A000005) with header pins to the main PCB at designator A1
2. Solder x8 680 Ω resistors (Digi-Key #CF14JT680RCT-ND) to the main PCB at designators R1, R2, R3, R4, R5, R6, R7, and R8
3. Solder x6 2 N3904 NPN transistors (Digi-Key #2N3904FS-ND) to the main PCB at designators Q1, Q2, Q3, Q4, Q5, and Q6
4. Solder x6 CL2N3-G 20 mA LED drivers (Digi-Key #CL2N3-G-ND) to the main PCB at designators U2, U3, U4, U5, U6, and U7
5. Solder x6 100 nF capacitors (Digi-Key #56-K104K20X7RK53H5G-ND) to the main PCB at designators C1, C2, C3, C4, C5, and C6
6. Solder x1 LM7805 5 V regulator (Digi-Key #296–47192-ND) to the main PCB at designator U1
7. Solder x10 2-pin JST headers (Digi-Key #455-B2B-EH-A-ND) to the main PCB at designators J1, J2, J4, J5, J6, J7, J8, J9, J10, and J11
8. Solder x1 4-pin JST header (Digi-Key #455-B4B-EH-A-ND) to the main PCB at designator J13
9. Solder x1 5-pin JST header (Digi-Key #455-B5B-EH-A-ND) to the main PCB at designator J12

### 5.3 Enclosure lower chamber

1. Secure the main PCB to the 3D-printed enclosure lower chamber (enclosure_lower_chamber.step) using four M3x12mm screws (McMaster-Carr #91292A114), 6 mm standoffs (McMaster-Carr #94669A101), and M3 hex nuts (McMaster-Carr #91828A211), and attach the 17 mm fan (Digi-Key #563–1108-ND) using three M2x14mm screws (McMaster-Carr #91292A835) and M2 hex nuts (McMaster-Carr #91828A111), as in Fig. 5a

**Fig. 5:**
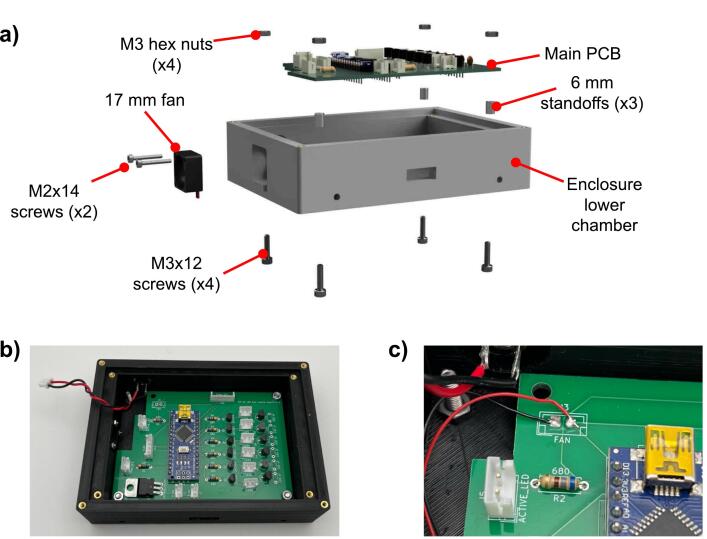
a) Exploded render of enclosure lower chamber assembly with components labeled, b) assembled lower box chamber, c) detail showing soldering of fan wires directly to PCB.
2. Cut two 100 mm sections of 24 AWG solid core wire and solder them to the power and ground terminals of the power jack (Amazon #B083W2YGXF)
3. Attach JST crimps (Digi-Key #455–1042-1-ND) to the ends of the power jack wires and insert them into a 2-position JST connector (Digi-Key #455–1000-ND)
4. Insert the power jack into the through-hole in the back of the lower enclosure chamber and secure it with the included hex nut, as in Fig. 5b
5. Attach the power input JST connector to its receptacle on the main PCB (P_IN, J2)
6. Solder the fan wires directly to the PCB at designator J3, as in Fig. 5c
7. Heat set x8 M2 threaded inserts (McMaster-Carr #94180A307) into the 3D printed 3 mm diameter holes where the mid plate and enclosure upper chamber will attach to the lower chamber
  a. Use a soldering iron set to 260 °C with a 2 mm diameter tip
  b. Place the tapered end of the threaded insert into the 3 mm diameter hole in the 3D printed enclosure lower chamber
  c. Insert the soldering iron into the hole on the other end of the threaded insert and press down until the top of the insert is flush with the 3D-printed surface
  d. Repeat for all x8 threaded inserts

### 5.4 LED columns

1. Solder x1 365 nm UV LED (Digi-Key #1125–1509-ND) to each of the x18 UV LED sub-boards at designator D1
2. Heat set x6 M2 threaded inserts (McMaster-Carr #94180A307) into the 3 mm diameter holes in the 3D printed LED column (led_column.step) using the method described above in section 5.3 in the locations shown in Fig. 4b
3. Once assembled, each LED circuit will consist of three LED sub-boards connected in series and connected to the main board via a JST connector as in Fig. 4c
4. Place x3 LED sub-boards with soldered LEDs into the 6.5 mm diameter holes in the 3D printed LED column and secure them using M2x4mm pan head screws (McMaster-Carr #92000A011)
5. Choose one of the LEDs to be designated as D1 per Fig. 4a, and designate the other two D2 and D3 as you continue clockwise around the LED column
6. Solder a 25 mm piece of wire between the “out” terminal of D1 and the “in” terminal of D2
7. Solder a 25 mm piece of wire between the “out” terminal of D2 and the “in” terminal of D3
8. Cut two 100 mm pieces of 24 AWG solid core wire and solder one to the “in” terminal of D1 and one to the “out” terminal of D3
9. Attach JST crimps (Digi-Key #455–1042-1-ND) to the other ends of the wires and insert them into a 2-position JST connector (Digi-Key #455–1000-ND)

### 5.5 User interface

1. Power switch
  a. Cut two 55 mm sections of 24 AWG solid core wire
  b. Solder one end of each wire to the terminals on the push-button power switch (Amazon #B0C2GBN5MX)
  c. Attach JST crimps (Digi-Key #455–1042-1-ND) to the other end of the wires and insert them into a 2-position JST connector (Digi-Key #455–1000-ND). The orientation of the wires in the connector does not matter
  d. Insert the power switch with soldered wires and JST connector into the 12.8 mm diameter hole in the 3D-printed controls housing (controls_housing.step). Secure the switch in place using the included hex nut, as in Fig. 6a

**Fig. 6:**
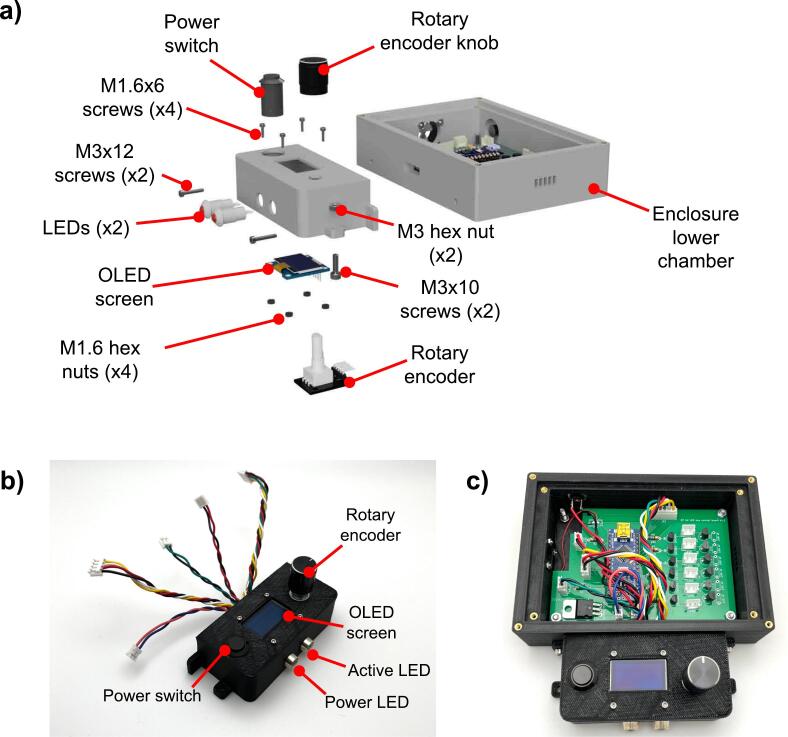
a) Exploded render of controls housing assembly with components labeled, b) assembled controls housing assembly showing component wires with JST connectors, c) controls housing attached to enclosure lower chamber with JST connectors attached to receptacles on PCB.
2. OLED screen
  a. Cut four 90 mm sections of 24 AWG solid core wire
  b. Solder one end of each wire to the terminals on the OLED screen (Amazon #B09C5K91H7)
  c. Attach JST crimps (Digi-Key #455–1042-1-ND) to the other end of the wires and insert them into a 4-position JST connector (Digi-Key #455–1002-ND). From left to right, the wires in the JST connector should be: GND, VCC, SCL, SDA
  d. Attach the OLED with soldered wires and JST connector to the 3D-printed controls housing using four M1.6x6mm screws (McMaster-Carr #91292A263) and M1.6 hex nuts (McMaster-Carr #90591A109), as in Fig. 6a
3. Rotary encoder
  a. Cut five 120 mm sections of 24 AWG solid core wire
  b. Solder one end of each wire to the terminals on the rotary encoder (Amazon #B07F26CT6B)
  c. Attach JST crimps (Digi-Key #455–1042-1-ND) to the other end of the wires and insert them into a 5-position JST connector (Digi-Key #455–1003-ND). From left to right, the wires in the JST connector should be: GND, VCC, SW, DT, CLK
  d. Remove the plastic knob from the encoder and pass the shaft through the 7.2 mm diameter hole in the 3D-printed controls housing. Secure the encoder to the housing using the included hex nut and restore the plastic knob onto the shaft, as in Fig. 6a
4. LEDs
  a. The system has two externally facing 5 mm LEDs: one green LED to indicate that the system is turned on and one red LED to indicate that the UV LEDs are active
  b. Install both LEDs into the LED bezels (Digi-Key #1568–1212-ND)
  c. Power (green) LED (Digi-Key #365–1181-ND)
    i. Cut two 95 mm sections of 24 AWG solid core wire
    ii. Solder one end of each wire to the two leads of the LED
    iii. Attach JST crimps (Digi-Key #455–1042-1-ND) to the other end of the wires and insert them into a 2-position JST connector (Digi-Key #455–1000-ND). From left to right, the wires in the JST connector should be: LED ground, LED forward voltage
  d. Active (red) LED (Digi-Key #365–1182-ND)
    i. Cut two 110 mm sections of 24 AWG solid core wire
    ii. Solder one end of each wire to the two leads of the LED
    iii. Attach JST crimps (Digi-Key #455–1042-1-ND) to the other end of the wires and insert them into a 2-position JST connector (Digi-Key #455–1000-ND). From left to right, the JST connector should be: LED ground, LED forward voltage
  e. Pass the power LED through the lefthand side 8 mm hole on the front of the 3D-printed controls housing, and pass the active LED through the righthand side 8 mm hole
  f. Secure both bezels to the 3D-printed controls housing with their included lock washers and hex nuts, as in Fig. 6a
5. Pass all JST connectors of the components through the 16 mm x 6 mm window in the rear of the controls housing. It is easiest to start with the 5-position and 4-position connectors first before passing the 2-position connectors through, see Fig. 6b
6. Pass all JST connectors through the 16 mm x 6 mm window on the front of the enclosure lower chamber
7. Attach the JST connectors to the corresponding JST receptacles on the main PCB, see Fig. 6c
  a. Power switch −> P_SW (J1)
  b. OLED screen −> OLED (J13)
  c. Rotary encoder −> Timer Ctrl (J12)
  d. Power LED −> PWR_LED (J4)
  e. Active LED −> ACTIVE_LED (J5)
8. Secure the controls housing to the enclosure lower chamber using two M3x12mm screws (McMaster-Carr #91292A114) and M3 hex nuts (McMaster-Carr #91828A211)
9. Place two M3x10mm screws (McMaster-Carr #91292A265) upside-down in the two flanges on each side of the controls housing and secure them with M3 hex nuts (McMaster-Carr #91828A211). These will be used as feet to balance the controls housing

### 5.6 Mid plate

1. Attach a small circular mirror (Amazon #B08X2W7MZM) to the center of each set of three 3 mm diameter holes on the laser cut acrylic mid plate, as in Fig. 7a

**Fig. 7:**
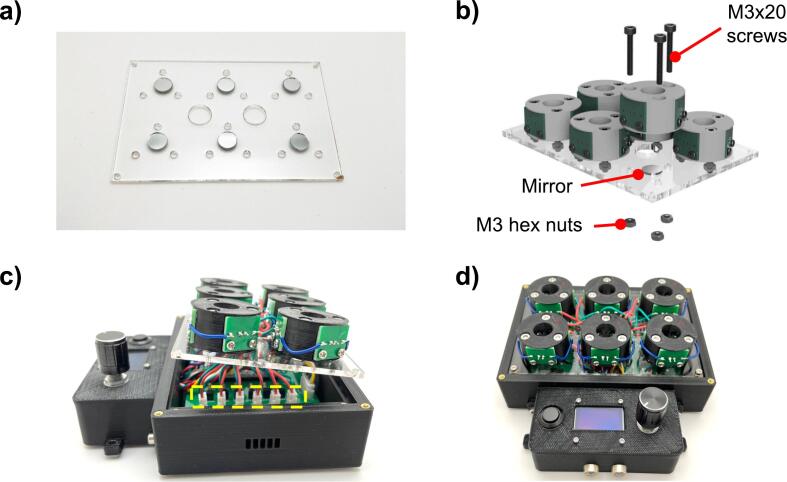
a) Laser cut midplate with adhesive-backed mirrors attached, b) exploded render of assembling UV LED columns onto midplate, c) attaching JST connectors from UV LED columns to their terminals on the main PCB (dashed yellow box), d) assembled midplate attached to enclosure lower chamber. (For interpretation of the references to colour in this figure legend, the reader is referred to the web version of this article.)
2. Secure all six LED columns onto the mid plate using M3x20mm screws (McMaster-Carr #91292A123) and M3 hex nuts (McMaster-Carr #91828A211), Fig. 7b
3. Pass the wires of each LED column through one of two 12 mm diameter holes on the mid plate
4. Hold the mid plate over the enclosure lower chamber and begin to plug each LED column JST connector into its respective JST receptacle on the main PCB, as shown in Fig. 7c
  a. Upper left −> LED1 (J6)
  b. Upper mid −> LED2 (J7)
  c. Upper right −> LED3 (J8)
  d. Lower left −> LED4 (J9)
  e. Lower mid −> LED5 (J10)
  f. Lower right −> LED6 (J11)
5. Secure the mid plate to the enclosure lower chamber using four M2x5mm screws (McMaster-Carr #91292A005) to thread into the previously installed M2 threaded inserts (McMaster-Carr #94180A307), see Fig. 7d

### 5.7 Polished aluminum tube sleeves

1. Cut six 25.5 mm sections of ½” diameter, 0.035″ wall thickness aluminum tubing (McMaster-Carr #89965 K491)
2. Polish the interior of the tube sections using successive grits of sandpaper
3. Clean the tube sections with acetone, isopropyl alcohol, and water
4. Place the tube sections in 0.505″ inner diameter, 0.745″ outer diameter aluminum washers (McMaster-Carr #94589A450)
5. Bond the tube sections to the washers using Loctite 11C adhesive (McMaster-Carr #1813A222), as shown in Fig. 8a

**Fig. 8:**
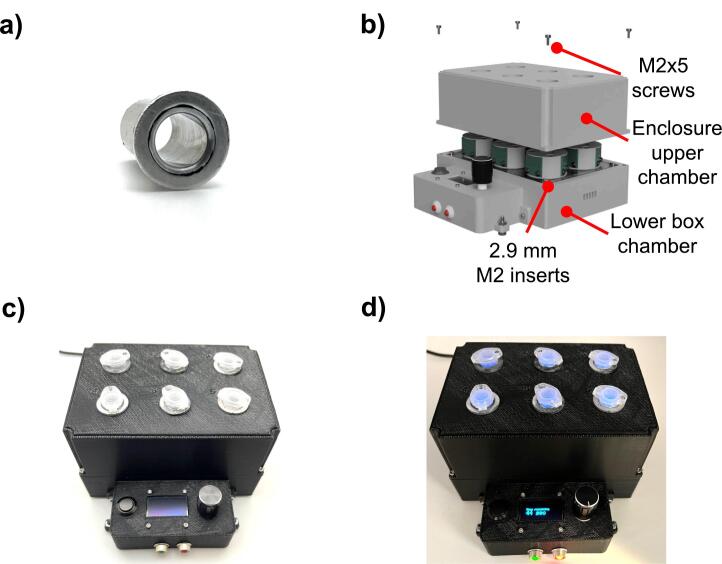
a) Polished aluminum tube sleeve, b) exploded render of enclosure upper chamber connecting to enclosure lower chamber, c) assembled system with 1.5 mL Eppendorf tubes, d) system performing UV LED treatment on 1.5 mL Eppendorf tubes.
6. Allow to cure overnight

### 5.8 Enclosure upper chamber

1. Attach the 3D-printed enclosure upper chamber (enclosure_upper_chamber.step) to the previously assembled enclosure lower chamber using four M2x5mm screws (McMaster-Carr #91292A005) to thread into the previously installed M2 threaded inserts (McMaster-Carr #94180A307), as shown in Fig. 8b
2. Place one polished aluminum tube sleeve in each of the six 15.5 mm diameter holes in the enclosure upper chamber, see Fig. 8c

## 6 Operation instructions

### 6.1 Safety

1. Do not operate the system with the enclosure upper assembly removed
2. Do not look directly down the LED column during processing due to risk of UV exposure

### 6.2 Tube loading

1. The system can accommodate up to six 1.5 mL Eppendorf tubes at a time
2. Place the desired number of Eppendorf tubes into the polished aluminum tube sleeves in the enclosure upper chamber
3. Be sure to place the tubes in the tube sleeves in increasing order as designated by the embossed digits on the enclosure upper chamber

### 6.3 Sample processing

1. Plug the 18 V power supply into the power receptacle on the back of the system
2. Turn on the system using the power switch on the controls housing
3. A welcome screen will be displayed on the OLED display
4. A menu will then be displayed asking the user to select the number of tubes to expose to LED light (between 1 and 6)
  a. Rotate the rotary encoder until the desired quantity of tubes to expose to LED light is displayed
  b. Click down on the rotary encoder knob to select this number
5. The exposure duration menu is displayed next, where the user can select the time (in seconds) of LED exposure
  a. Rotate the rotary encoder until the desired exposure time is displayed
  b. Click down on the rotary encoder knob to select this number
6. The system will begin processing, and will display the number of seconds left in the recipe on the OLED display, as shown in Fig. 8d

## 7 Validation and characterization

### 7.1 Characterization of the UV LED system

**Fig. 9:**
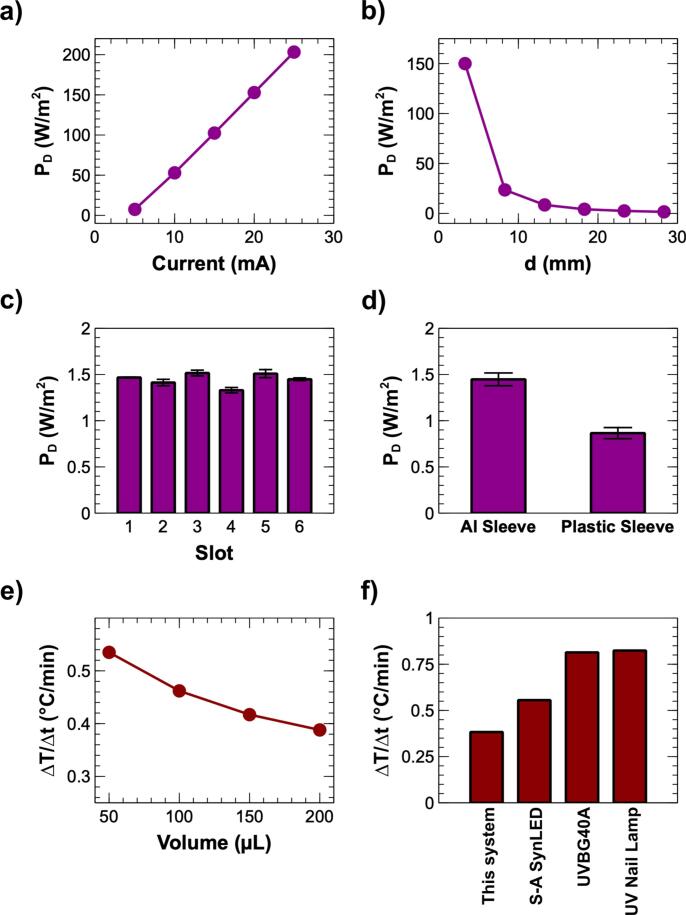
a) Power density of a single led versus applied current, measured at a distance of 3.3 mm from the bulb, b) power density of a single LED versus distance at a forward current of 20 mA, c) Power output of each slot of the box as measured from the top of the aluminum tube sleeve at a forward current of 20 mA, d) comparison of achievable power density when using the polished aluminum sleeve versus a 3D-printed plastic sleeve, e) average rate of temperature change versus sample volume over 10 min with a forward current of 20 mA, f) comparison of rates of temperature change over 10 min for commercial alternatives (volume = 150 µL).

Low power UV LEDs were selected for this system to provide efficient photoconjugation with minimal sample heating. These LEDs have a peak wavelength of 365 nm and a nominal power output of 5.5 mW at 25 mA forward current. An optical power meter (Thorlabs PM100D) with a photodiode sensor (Thorlabs S170C) was used to measure the optical power density of the LEDs. A power curve measured 3.3 mm from the LED bulb (the distance between the LEDs and the Eppendorf tube in the system) is shown in Fig. 9a. The output power density (P_D_) peaks at just over 200 W/m^2^ at 25 mA, the maximum forward current rating of the LEDs. To reduce stress on the LEDs and lower the amount of heat put into the Eppendorf tubes, 20 mA current regulators were chosen resulting in an output power density of 152 W/m^2^ per LED at 3.3 mm (with 3 LEDs this translates to a power density of 456 W/m^2^ per tube). The power density drops off steeply with distance from the source as shown in Fig. 9b, so it was necessary to keep the bottom of the Eppendorf tube held centered at a minimum distance between all three LEDs for efficient photoconjugation. Fig. 9c shows the power reading for each slot measured from the top of the aluminum tube sleeve. The power density here is greatly reduced from the 456 W/m^2^ seen at the bottom of the tube due to the 90° angle between the LED cone and the power meter − here the UV light can only reach the power meter after reflecting off the mirror and polished aluminum tube sleeve. A very low slot-to-slot variability is observed (standard deviation of ∼ 0.07 W/m^2^). To demonstrate the importance of the polished aluminum tube sleeve, an equivalent plastic version of the sleeve was 3D-printed out of PLA. A comparison of power density achieved when using each material is shown in Fig. 9d, with the polished aluminum sleeve offering a ∼ 67% improvement over the plastic version.

The average temperature increase versus sample volume is shown in Fig. 9e. The measurements were obtained by placing the corresponding volume of DI water into a 1.5 mL Eppendorf tube with a K-type thermocouple and recording the temperature for 10 min through a Pico USB TC-08 data logger. As expected, lower sample volumes result in higher heating rates due to a smaller thermal mass to absorb energy input into the liquid by the UV LEDs. A comparison of the temperature increase rate in this system to available commercial options is shown in Fig. 9f. Here, 150 µL of DI water was loaded into a 1.5 mL and again monitored using a submerged K-type thermocouple. This system showed the lowest temperature change rate of all systems tested, demonstrating the ability of low-power LEDs to reduce process temperatures. An extended experiment of a 150 µL volume treated over 40 min showed that this system resulted in a < 10 °C increase in temperature, while the UVBG40A wand produced a > 20 °C increase (see Supplementary Fig. 3).

### 7.2 Photocrosslinking of secondary nanobodies to primary antibodies

**Fig. 10:**
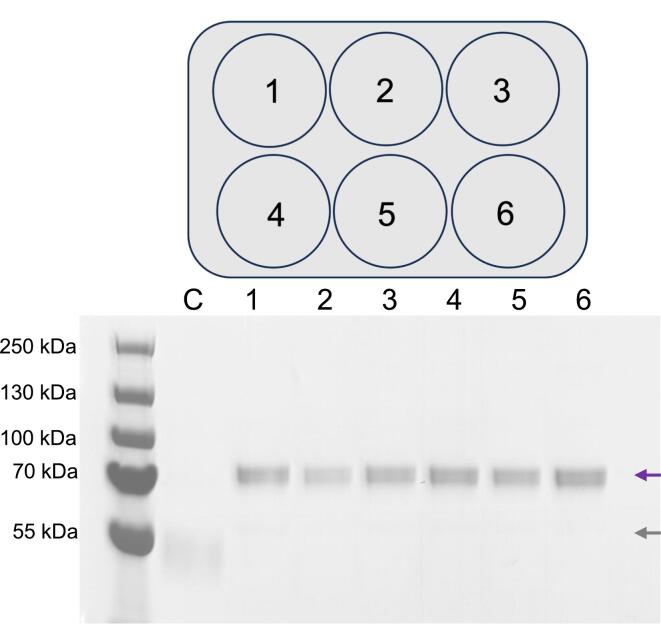
SDS-PAGE reducing gel showing complete crosslinking of nanobodies (15 kDa) to antibody heavy chain across all slots (1–6) using the UV LED system after a 5-minute photocrosslinking process. The purple arrow indicates the band corresponding to the conjugated heavy chain fragment of the antibody (1 µg per well), with the size shifting to 70 kDa (a 15 kDa increase), while the gray arrow marks the bands of the unconjugated heavy chain fragment of the antibody (C, control lane, 50 kDa).

As mentioned previously, the UV LED system was designed to address the needs and challenges in current photoreactive bioconjugation applications. Specifically, we applied it for the rapid photocrosslinking of secondary nanobodies (NBs) to antibodies in standard 1.5 mL Eppendorf tubes (see Supplementary Fig. 2). To assess the system’s capacity and functionality, we performed an experiment where each slot of the system contained a 1.5 mL Eppendorf tube with 10 µg of antibodies and an excess of NBs, which were engineered with a photoreactive moiety that can be activated for covalent conjugation to antibodies upon UV irradiation. To evaluate the conjugation efficiency, we performed SDS-PAGE analysis, a standard method for separating proteins based on their molecular weight. As shown in the SDS-PAGE result (Fig. 10), a clear upward shift in molecular weight was observed for the conjugated heavy chain fragment of the antibodies. All six slots exhibited consistent conjugation efficiency.

**Fig. 11:**
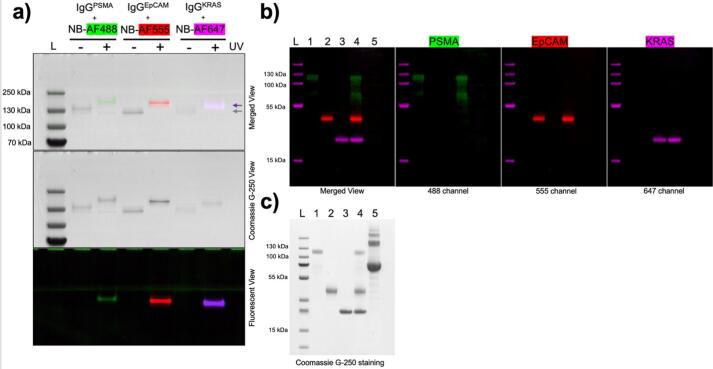
Validation of UV LED system for efficient photocrosslinking without quenching fluorophores in immunofluorescence applications. a) SDS-PAGE analysis of 1 µg primary antibodies (IgG) conjugated with nanobodies (NB) labeled with AF488 (green), AF555 (red), and AF647 (purple) using the UV LED system. The top panel (Merged View) shows the conjugation of fluorescently labeled nanobodies to primary antibodies with molecular weight upward shifts (indicated by purple arrows) corresponding to the successful conjugates (IgG + NBs). The middle panel (Coomassie G-250 View) confirms the presence of the whole IgG or the successful conjugates. The bottom panel demonstrates the preservation of fluorescence signals from the nanobodies in their respective fluorescent channels, indicating successful photoconjugation without compromising fluorophore integrity. b) Multiplexed fluorescent western blotting of three oncogenic protein markers using their corresponding conjugated antibodies labeled with AF488, AF555, and AF647. The lanes marked 1 to 5 represent: lane 1, loaded with PSMA only (expected size 82 kDa, the higher molecular weight observed is due to glycosylation, as is common for membrane proteins); lane 2, loaded with EpCAM only (expected size 30 kDa); lane 3, loaded with KRAS only (expected size 22 kDa); lane 4, a mixture of the three protein markers; and lane 5, a BSA control. c) Coomassie G-250 staining of the SDS-PAGE gel with the same sample loading setup as in the fluorescent WB above, confirming the molecular weight of individual protein markers, the mixture, and the BSA control. (For interpretation of the references to colour in this figure legend, the reader is referred to the web version of this article.)

We further evaluated the compatibility of the UV LED system for photoconjugation by determining its ability to preserve the integrity of fluorophores for downstream fluorescence molecular imaging in biomedical applications. Three 1.5 mL Eppendorf tubes, each containing 1 µg of primary antibodies targeting three known oncogenic markers (PSMA, EpCAM, and KRAS), were incubated with secondary nanobodies at a molar ratio of 1:3 (antibody:nanobody) for a complete conjugation, each individually labeled with 0.3 µg AF488, AF555, and AF647 labeled nanobodies. This setup represents the minimal amount typically required for antibody labeling in immunofluorescent labeling, allowing us to fully evaluate the UV LED system’s compatibility in minimizing fluorophore quenching in practical applications. After a 5-minute photocrosslinking process, the SDS-PAGE results (Fig. 11a) show that UV exposure led to the complete conjugation of AF488, AF555, and AF647-labeled nanobodies to their corresponding primary antibodies, with clear upward shifts in molecular weight, as indicated by the purple arrows. The corresponding Coomassie G-250 stain (middle panel) confirms the presence of the whole primary antibodies, while the fluorescent views (bottom panel) display strong, distinct fluorescence signals in the green, red, and purple channels, respectively. This indicates that the UV LED system provides sufficient capability for efficient photoconjugation while maintaining the fluorescence integrity of fluorophore on the nanobodies.

Following the photocrosslinking process above, we further assessed the ability of the conjugated antibodies in multiplexed western blotting. The UV-irradiated, fluorophore-labeled antibodies (AF488, AF555, and AF647) were used to probe for three oncogenic proteins PSMA, EpCAM, and KRAS. The results showed that, after UV exposure, the conjugated antibodies retained their specificity and effectively bound to their target proteins (Fig. 11b). The distinct fluorescence signals in the respective channels (green, red, and purple) confirmed the successful detection of these oncogenic markers, demonstrating that the UV LED system not only facilitates efficient photocrosslinking but also preserves the functional integrity and specificity of the antibodies for multiplexed immunolabeling. The observed multiplexed immunolabeling results are consistent with the standard SDS_PAGE analysis which shows the molecular weight of these recombinantly expressed oncogenic proteins, along with the mixture and the BSA control (Fig. 11c).

**Fig. 12:**
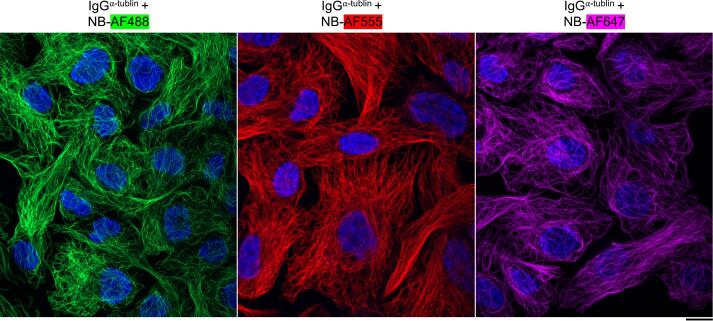
Immunofluorescence imaging of microtubule cytoskeleton in U2OS cells. The α-tubulin antibodies were individually conjugated with nanobodies in AF488 (green), AF555 (red), and AF647 (purple) using the UV-LED system. Nuclei are stained with DAPI (blue). Scale bar = 10 µm. (For interpretation of the references to colour in this figure legend, the reader is referred to the web version of this article.)

We then validated that the α-tubulin antibody conjugated through the UV LED system can be used for efficient immunofluorescent labeling in U2OS cells. As shown in Fig. 12, the microtubule cytoskeleton is specifically labeled, with clear and distinct fluorescence observed in the respective channels for each fluorophore: AF488 (green), AF555 (red), and AF647 (purple). This suggests the UV LED system is well-suited for conjugating antibodies with fluorescently labeled nanobodies for immunofluorescent applications. This further confirms that the UV LED system is fully compatible with the photocrosslinking bioconjugation of our fluorescently labeled nanobodies to a primary antibody for subsequent use in immunofluorescence applications.

### 7.3 Conclusion

In this work, we have demonstrated methods for assembling a low-cost UV LED exposure system for photochemistry applications using standard rapid prototyping techniques such as 3D-printing, laser cutting, and basic electronics. The system employs low-power 365 nm LEDs to achieve efficient conjugation with minimal temperature increase compared to mercury lamp-based UV sources, allowing for extended processing times without the risk of denaturing or photobleaching samples. To validate the system, we performed experiments measuring the UV LED intensity and temperature increase under various conditions. The rapid and efficient nature of the system underscores its suitability for antibody conjugation, making it an ideal tool for high-throughput applications that require fast and reliable conjugation in antibody bioengineering. To this end, we demonstrated the ability to conjugate nanobodies to IgG antibodies for subsequent immunofluorescent imaging using the system, highlighting its capacity to preserve fluorescence post-conjugation. Beyond photocrosslinking-based conjugation, we also see the controlled and uniform UV irradiation provided by this system, having potential utility for photocleavage-based applications in biomedical research. Beyond nanobody conjugation, this system has potential applications in photochemistry, purification, and UV exposure testing. Future improvements to this system could include expanding the number of samples processed at once by adding additional slots, using a larger fan to achieve even lower process temperatures, and electropolishing of aluminum tube sleeves for increased UV irradiation throughout the volume of the tube.

## Ethics statements

No human or animal subjects were involved in the presented work.

## CRediT authorship contribution statement

**Samuel Olson:** Writing – original draft, Visualization, Validation, Software, Methodology, Investigation, Conceptualization. **Gulsu Sener:** Writing – review & editing, Data curation. **Alan Weisgerber:** Writing – review & editing. **Kai Tao:** Writing – review & editing, Project administration, Funding acquisition, Data curation, Conceptualization.

## Declaration of competing interest

The authors declare that they have no known competing financial interests or personal relationships that could have appeared to influence the work reported in this paper.
