# Supplementary material for: A programmable benchtop photocrosslinking chamber for controlled bioconjugation
Source: HardwareX. 2026 Jun 15;27:e00804. doi: 10.1016/j.ohx.2026.e00804 (PMC13293757; doi:10.1016/j.ohx.2026.e00804)
Supplement: Supplementary Data 1 [file mmc1.docx]

**Supplementary Information for**

**A programmable benchtop photocrosslinking chamber for controlled bioconjugation**

**Antibody-nanobody labeling instructions**

1. **Sample preparation**
   1. Mix 0.3 µg of secondary nanobodies with 1 µg of target antibody (molar ratio ~3:1 nanobody:antibody) in PBS(−), with a total reaction volume up to 100 µL, in a standard 1.5 mL Eppendorf microcentrifuge tubes. Mix thoroughly by gentle pipetting.
   2. **Leave the mixture on a rocker at 500 rpm at RT for 5 minutes.**
2. **Photo-crosslinking**
   1. After the incubation above, place the Eppendorf tubes into this UV photoreactor.
   2. Be sure to place the tubes in the tube sleeves in increasing order as designated by the embossed digits on the enclosure upper chamber.
   3. Plug the 18V power supply into the power receptacle on the back of the system.
   4. Turn on the system using the power switch on the controls housing, a welcome screen will be displayed on the OLED display.
   5. A menu will then be displayed asking the user to select the number of tubes to expose to LED light (between 1 and 6), rotate the rotary encoder until the desired quantity of tubes to expose to LED light is displayed.
   6. Click down on the rotary encoder knob to select this number.
   7. **
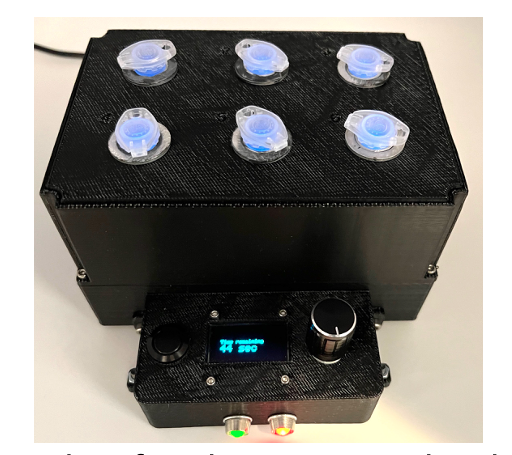
** The exposure duration menu is displayed next, where the user can select the time (in seconds) of LED exposure. Rotate the rotary encoder until the desired exposure time is displayed.
   8. Click down on the rotary encoder knob to select this number.
   9. The system will begin processing, displaying the number of seconds left in the recipe on the OLED display, as shown in Figure below.

**Supplementary Figure 1**: The system performing UV LED treatment on 1.5 mL Eppendorf tubes.


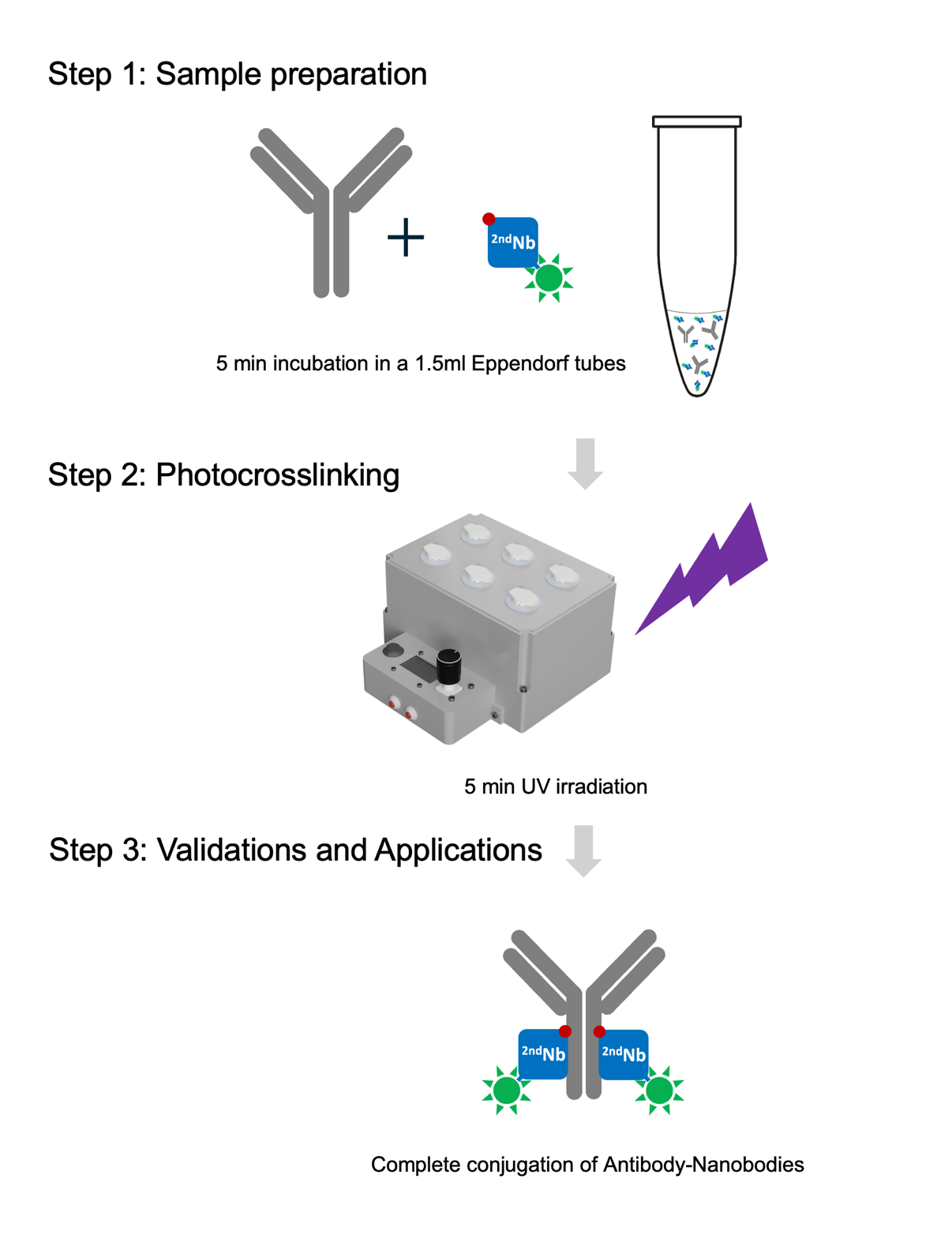


**Supplementary Figure 2**: Schematic illustration of antibody–nanobody photocrosslinking using the programmable benchtop UV chamber. Red dots indicate a functional photocrosslinker on the nanobodies, the green star represents the fluorophores. Step 1: Sample preparation, antibodies are incubated with these engineered secondary nanobodies in a 1.5 mL Eppendorf microcentrifuge tube for 5 minutes to allow their molecular interactions. Step 2: Photocrosslinking, the reaction mixture is exposed to controlled UV irradiation (365 nm) for 5 minutes using the programmable benchtop chamber, enabling covalent crosslinking between the nanobody and antibody. Step 3: Validations and applications, the resulting antibody-nanobody conjugates are generated and can be used for downstream applications such as immunofluorescence imaging.


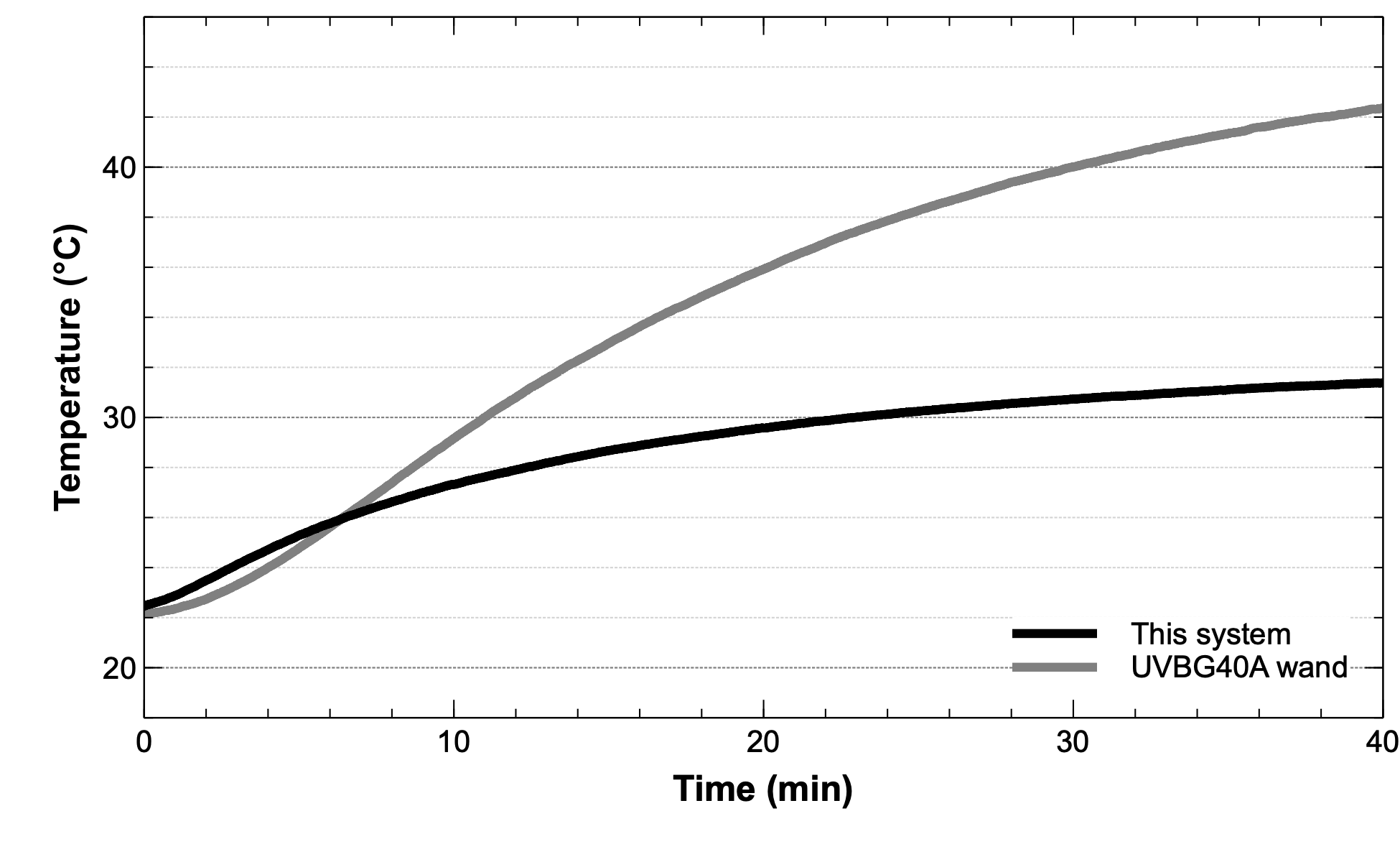


**Supplementary Figure 3**: Temperature change of deionized water (150 µL in 1.5 mL Eppendorf tube) treated over 40 minutes in this system vs. a UVBG40A mercury lamp wand, as measured via a K-Type thermocouple submerged in the liquid
